# Genetic Characterization of Dilated Cardiomyopathy in Romanian Adult Patients

**DOI:** 10.3390/ijms25052562

**Published:** 2024-02-22

**Authors:** Oana Raluca Voinescu, Bogdana Ioana Ionescu, Sebastian Militaru, Andreea Sorina Afana, Radu Sascau, Laura Vasiliu, Sebastian Onciul, Mihaela Amelia Dobrescu, Ramona Alina Cozlac, Dragos Cozma, Raluca Rancea, Bogdan Dragulescu, Nicoleta Ioana Andreescu, Maria Puiu, Ruxandra Oana Jurcut, Adela Chirita-Emandi

**Affiliations:** 1 Department of Cardiology, Cardiology Discipline II, University of Medicine and Pharmacy “Victor Babeș”, Eftimie Murgu Sq., 300041 Timișoara, Romaniaalina-ramona.cozlac@umft.ro (R.A.C.); dragos.cozma@umft.ro (D.C.); 2 Department of Cardiology, University of Medicine and Pharmacy ‘Carol Davila’, Dionisie Lupu Street, no. 37, Sector 2, 4192910 Bucharest, Romania; 3 Expert Center for Rare Cardiac Genetic Diseases, Emergency Institute for Cardiovascular Diseases ‘Prof.dr.C.C.Iliescu’, Fundeni 258, 022328 Bucharest, Romania; 4 Department of Cardiology, Craiova University of Medicine and Pharmacy, Petru Rareș Street no 2, 200349 Craiova, Romaniaandreea.afana@gmail.com (A.S.A.); 5 Cardiomed Hospital, Craiova, Str. Spania, Nr. 35A, 200513 Craiova, Romania; 6 Internal Medicine Department, “Grigore T. Popa” University of Medicine and Pharmacy, 16 Universitatii Street, 700503 Iași, Romania; 7 Cardiology Department, Cardiovascular Diseases Institute “Prof. Dr. George I. M. Georgescu”, 50 Boulevard Carol I, 700503 Iași, Romania; 8 Genetics Department, Craiova University of Medicine and Pharmacy, Petru Rareș 2 Street, 200349 Craiova, Romania; 9 Regional Centre of Medical Genetics Dolj, Emergency County Hospital Craiova, 200642 Craiova, Romania; 10 Cardiology Department, Institute of Cardiovascular Diseases, Gheorghe Adam Street, 13A, 300310 Timișoara, Romania; 11 Cardiology Department, Heart Institute Niculae Stăncioiu, 19–21 Motilor Street, 400001 Cluj-Napoca, Romania; 12 Communications Department, Politehnica University Timisoara, sq Victoriei 2, 300006 Timișoara, Romania; bogdan.dragulescu@upt.ro; 13 Department of Microscopic Morphology, Genetics Discipline, Center of Genomic Medicine, University of Medicine and Pharmacy “Victor Babeș” Timișoara, 2 Piaţa Eftimie Murgu Street, 300041 Timişoara, Romania; 14 Regional Center of Medical Genetics Timiș, Clinical Emergency Hospital for Children “Louis Țurcanu” Iosif Nemoianu Street N°2, 300011 Timișoara, Romania

**Keywords:** dilated cardiomyopathy, heart failure, genetic testing, next-generation sequencing

## Abstract

Dilated cardiomyopathy (DCM) represents a group of disorders affecting the structure and function of the heart muscle, leading to a high risk of heart failure and sudden cardiac death (SCD). DCM frequently involves an underlying genetic etiology. Genetic testing is valuable for risk stratification, treatment decisions, and family screening. Romanian population data on the genetic etiology of DCM are lacking. We aimed to investigate the genetic causes for DCM among Romanian adult patients at tertiary referral centers across the country. Clinical and genetic investigations were performed on adult patients presenting to tertiary hospitals in Romania. The genetic investigations used next-generation sequencing panels of disease-associated DCM genes. A total of 122 patients with DCM underwent genetic testing. The mean age at DCM diagnosis was 41.6 ± 12.4 years. The genetic investigations identified pathogenic or likely pathogenic variants in 50.8% of participants, while 25.4% had variants of unknown significance. Disease-causing variants in 15 genes were identified in people with DCM, with 31 previously unreported variants. Variants in *TTN*, *LMNA*, and *DSP* explained 75% of genetic causes for DCM. In total, 52.4% of patients had a family history of DCM/SCD. Left ventricular ejection fraction of <35% was observed in 41.9% of patients with disease-causing variants and 55% with negative or uncertain findings. Further genotype-phenotype correlations were explored in this study population. The substantial percentage (50.8%) of disease-causing variants identified in patients with DCM acknowledges the importance of genetic investigations. This study highlights the genetic landscape in genes associated with DCM in the Romanian population.

## 1. Introduction

Dilated cardiomyopathy (DCM) (ORPHA CODE 217604) is a myocardial disease characterized by the presence of left ventricular dilatation with localized or diffuse hypokinesia, not solely explained by abnormal loading conditions (e.g., hypertension and valvular heart disease) or coronary artery disease [1], leading to a high risk of heart failure (HF) and sudden cardiac death (SCD). It has an estimated reported prevalence of 0.036–0.4% in the general population and represents the leading cause for heart transplantation [1,2]. DCM frequently involves an underlying genetic etiology, identified in approximately 40% of cases [3,4]. The genetic heterogenity underlying DCM makes DCM the most genetically complex of all inherited cardiomyopathies. Modern testing options have found that DCM has one dominant genetic background in around 20% of patients, titin mutations (TTN), allowing research to focus on a subset of this disease. It is also possible to identify combinations of genetic mutations that contribute to the disease. The outcome with the same causative variant seems to be significantly influenced by sex or epigenetic factors and second hit factors (like alcohol consumption, pregnancy, or cardiotoxic agents), but also by the presence of additional genetic variants. These findings provide a basis for the development of both existing and novel therapeutic approaches targeted at the specific genetic pathways implicated in DCM [5]. Genetic testing using next-generation sequencing (NGS) is valuable for risk stratification, treatment decisions, and family screening. The recent Expert Consensus Statement on the state of genetic testing for cardiac diseases, including DCM, provides the diagnostic criteria for patient selection [6]. NGS has become more accessible, however, in Romania, genetic testing is restricted by limited resources, and sometimes involves patient’s financial contributions. While detailed geographical mapping of the genetic landscape is of great interest by describing founder mutations as well as disease prevalence at a country level [7], population data regarding the genetic etiology of DCM in Romanian people are lacking.

We aimed to investigate the diagnostic yield of genetic testing for DCM among Romanian adult patients at tertiary referral centers across the country. Furthermore, we aimed to investigate differences between those with significant genetic findings (disease-causing variants) versus those with negative results, in order to understand if patient selection could be improved in a setting with limited resources. Also, we aimed to evaluate the gene phenotype correlations in Romanian people with DCM to help improve clinical management. Potentially, a selection of genes with higher prevalence in the Romanian population could lead to more focused and cost-effective gene testing for DCM.

## 2. Results

### 2.1. Clinical Characteristics and Gene–Phenotype Correlations

A total of 122 Romanian adult patients with DCM, predominantly men (66.6%), received genetic testing using NGS panels for cardiomyopathies (number of genes included ranged between 54 and 179). The main clinical patient characteristics, a comparison between those with disease-causing versus negative findings in genetic testing, and a comparison between familial versus sporadic cases are presented in Table 1. The mean age at DCM diagnosis was 41.6 ± 12.4 years. Heart failure symptoms led to diagnoses in the majority of the patients, 101 (82.8%), while 3 (2.4%) people presented with cardiac arrest and only 6.5% were incidentally diagnosed, due to routine evaluation or family screening. More than half of the participants (52.4%) had a family history of DCM or SCD. An LVEF of <35% was observed in (48.4%) of people with DCM. Almost one-third (30.3%) of patients with DCM had a history of ventricular arrhythmia, while 39.3% had an implanted device (pacemaker, ICD, CRTD, or CRTP). One-third (33.3%) of people with DCM had conduction abnormalities represented by an atrioventricular block or intraventricular conduction disorders. Seventy-three (59.8%) participants were investigated using cardiac magnetic resonance with a high rate for identification of late gadolinium enhancement (fibrosis on CMR) in almost three-quarters of participants (73.9%).

The main clinical characteristics and imagistic findings of the patients with versus without disease-causing variants (pathogenic and likely pathogenic) (Table 1) were not statistically different, except for atrial fibrillation, which was more prevalent in people with disease-causing variants (25.8% vs. 11.6%).

When comparing familial versus sporadic disease presentation (Table 1), significantly more females were identified in the familial versus the sporadic DCM group (45.3% vs. 22.4%). Additionally, age of diagnosis and age at follow-up were significantly lower in the familial group. The reason for presentation was significantly different between groups, with more people presenting due to symptoms in the sporadic group.

The comparison between males and females in the 122 people with DCM did not show statistically significant differences in the variables assessed.

### 2.2. Genetic Characterization

All patients underwent genetic testing for DCM. In 60/122 (49.2%) of participants, genetic testing was performed using commercially available panels, ranging from 54 to 179 genes, chosen at the discretion of the attending cardiologist or genetic counselor (Invitae). Another 15/122 (12.3%) underwent familial screening based on sequencing the target genes found in the related proband (Invitae). The other DCM patients, 47/122 (38.5%), had genetic testing performed in the Genomic Center of the University of Medicine and Pharmacy “Victor Babes” Timisoara, using a panel of 174 genes (TruSightCardio Illumina).

**Figure 1:**
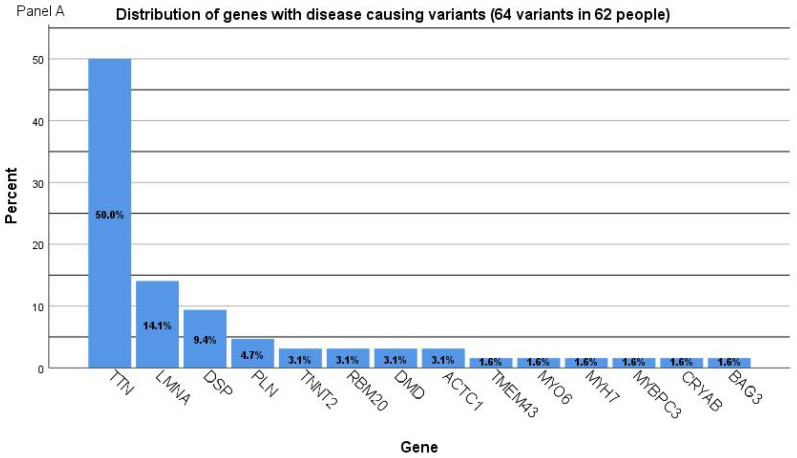

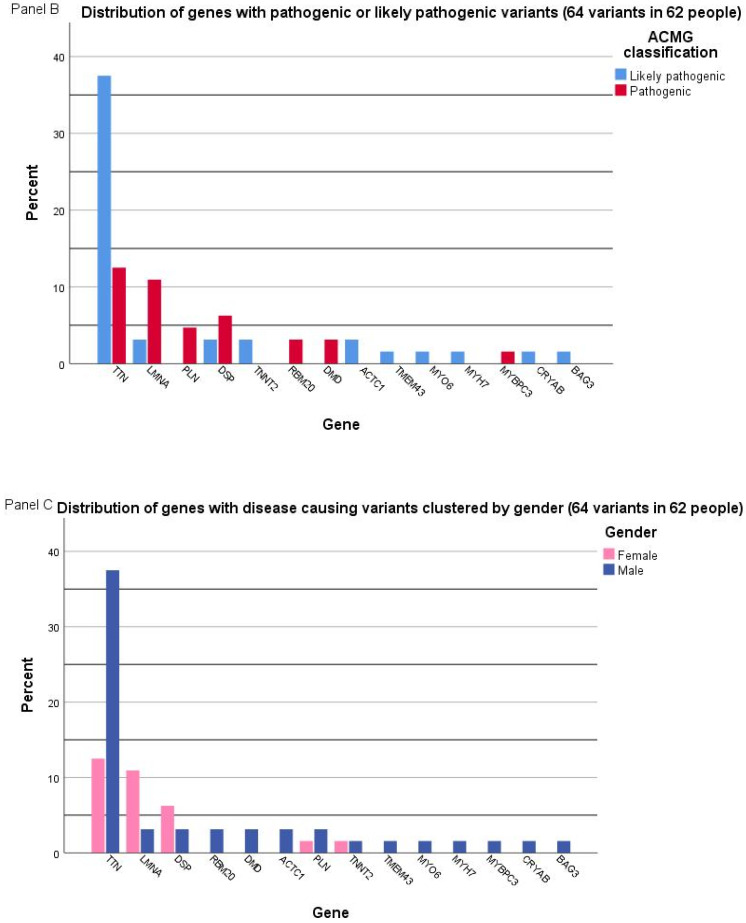
(Panel **A**) Distribution of genes with disease-causing (pathogenic and likely pathogenic) variants in people with DCM from Romania (64 variants in 62 people). (Panel **B**) Distribution of genes with pathogenic and likely pathogenic variants identified in people with DCM. (Panel **C**) Distribution of genes with disease-causing variants clustered by gender.

Genetic investigations identified pathogenic or likely pathogenic variants in 62/122 (50.8%) participants (41 males, 66.1%). Disease-causing variants in 15 genes were identified in people with DCM in the following genes: *TTN* (MIM 118840), *LMNA* (MIM 150330) *DSP* (MIM 125647), *TNNT2* (MIM 191045), *RBM20* (MIM 613171), *PLN* (MIM 172405), *DMD* (MIM 300377), *ACTC1* (MIM 102540), *TMEM43* (MIM 612048), *MYO6* (MIM 600970), *MYH7* (MIM 160760), *MYBPC3* (MIM 600958), *CRYAB* (MIM 123590), and *BAG3* (MIM 603883). The distribution of pathogenic or likely pathogenic variants is presented in Figure 1. Variants in *TTN*, *LMNA*, and *DSP* explained 75% of the genetic causes for DCM. Among the disease-causing variants identified, 18 were missense, 20 were nonsense, 25 were frameshift indels, and 2 were splice-site (Table 2). Thirty-one disease-causing variants (50%) were novel/previously unpublished (Table 2). Of 32 variants (all truncating) in the *TTN* gene, 18 variants were frameshift, 13 were nonsense (stopgain), and 1 was splice-site. In the *LMNA* gene, four variants were truncating (two frameshift and two nonsense), while five variants were missense. Particular discoveries included: two people with DCM had two significant variants associated with DCM (Table 2). One person had incidental findings related to the *RYR1* gene. One patient had a homozygous *PLN* variant. Variants of unknown significance are presented in Supplementary Table S1.

### 2.3. Genotype–Phenotype Correlation

When comparing patients with *TTN* versus *LMNA* variants, there were significantly more females in the *LMNA* group (Table 3). Heart conduction disorders, atrial fibrillation, and device implantation were significantly more prevalent in people with *LMNA* variants compared to those with *TTN* variants. All participants (8/8) with *LMNA* variants that performed CMR had fibrosis, compared to 76.2% (16/21) in *TTN*, however, this difference was not statistically significant.

**Figure 2:**
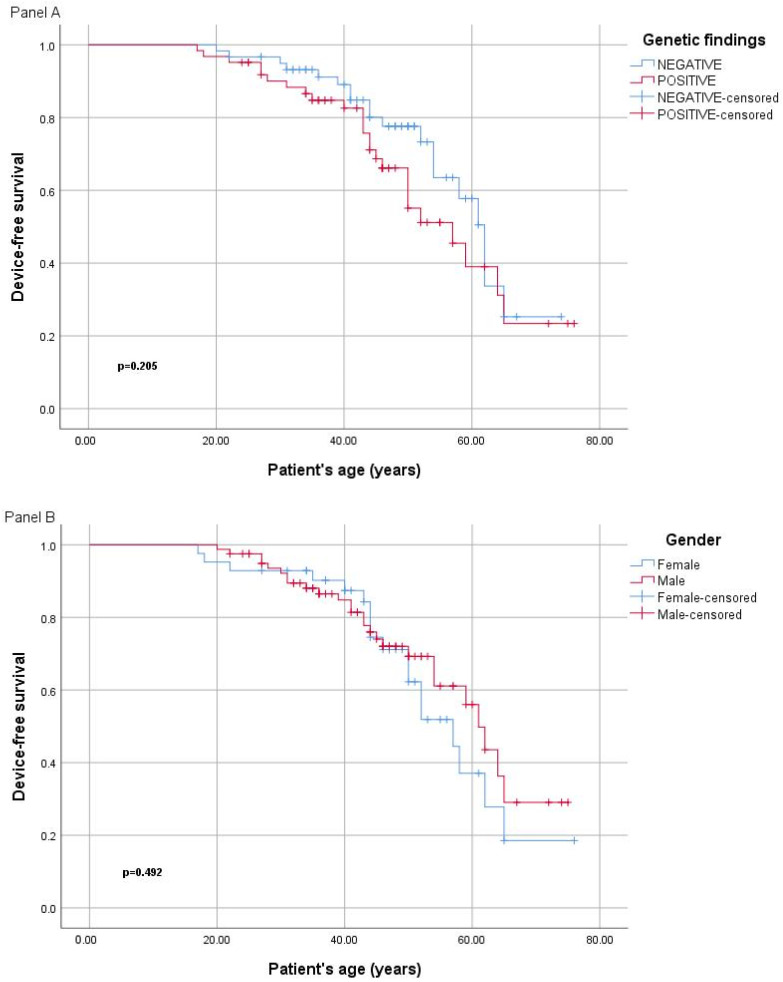

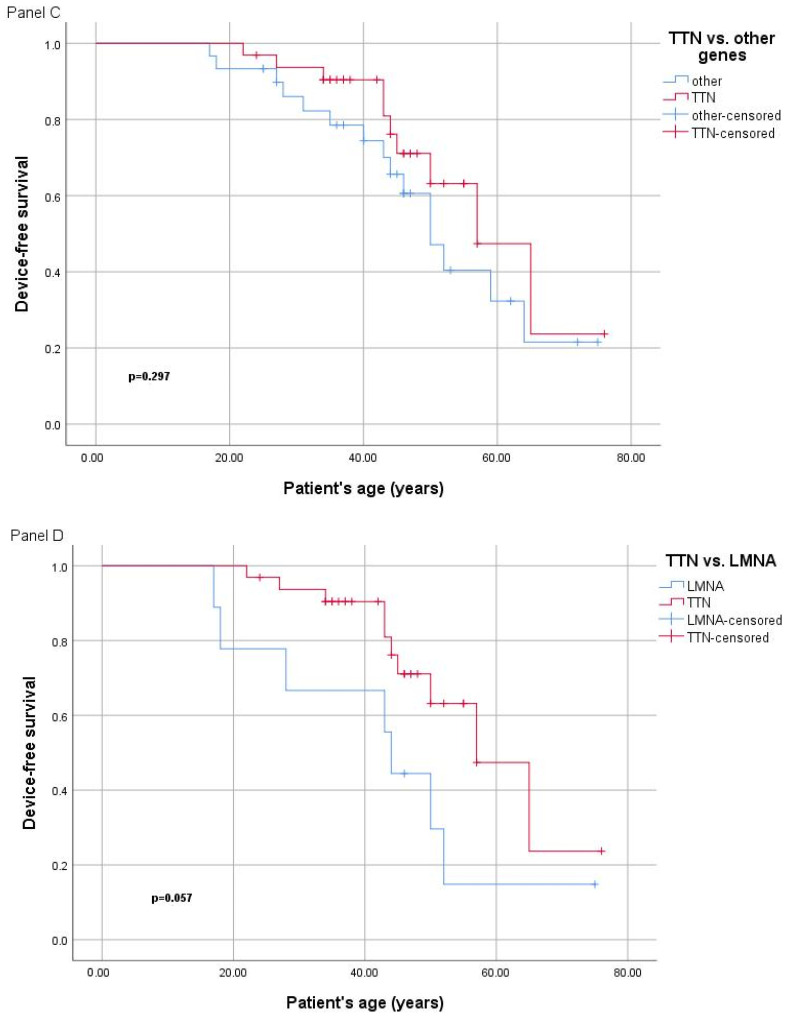
(Panel **A**) Device free survival curves (Kaplan–Meier) in people with DCM with disease-causing variants versus those without (panel **A**), female versus males (panel **B**), those with variants in *TTN* versus those with findings in other genes (panel **C**), and those with variants in *TTN* versus *LMNA* genes (panel **D**).

Heart transplantation was performed in one patient with an *LMNA* variant. No significant differences were observed when evaluating the disease severity between the four patients with truncating variants in *LMNA* and the five with missense variants. Five people were deceased in the present cohort, three of which had disease-causing variants identified (one in *BAG3* and two in *TTN*). There were significantly lower BNP (brain natriuretic peptides) values in people with *TTN* variants compared to other genes; however, the number of people tested is too small to enable accurate interpretation. A comparison between device-free survival curves in different groups is shown in Figure 2. The comparison included: (1) patients with DCM with disease-causing variants versus those without, (2) females versus males, (3) those with variants in *TTN* versus those with findings in other genes, and (4) those with variants in *TTN* versus *LMNA* genes.

### 2.4. Particular Cases in Regards to Molecular Genetics Findings

In this cohort, two people had more than one disease-causing variant, both males (Table 2). One patient had a pathogenic *PLN* variant associated with a likely pathogenic *TTN* variant, each with a heterozygous status. He was diagnosed at age 44 years, had severely decreased LEVF, and had an implantable cardioverter-defibrillator. Another patient was diagnosed with pathogenic heterozygous variants in *MYBC3* and *PLN*. Their clinical picture involved severely impaired cardiac function, a history of significant arrhythmias, and cardioverter-defibrillator implantation. One other female patient presented with homozygous *PLN* variants. She was diagnosed with DCM at age 30 years and had LVEF of 23% at age 37 years, and was thus considered to have severe DCM. One patient had an incidental discovery of an *RYR1* variant; however, no significant personal history was reported from the *RYR1* spectrum. In addition, one patient was identified with a likely pathogenic CRYAB variant, with a clinical picture of multisystemic disease, including myopathy affecting the proximal girdle and limb muscles, with moderate systolic dysfunction, atrial fibrillation, and permanent pacemaker stimulation for sinus node dysfunction.

## 3. Discussion

This is the first study that investigated the molecular background of a Romanian DCM patient cohort evaluated in several tertiary cardiology hospitals. Our study population had comparable clinical characteristics to other DCM cohorts previously published [8]. The INHERITANCE European study (where Romania did not participate) was a multi-center, multi-national (8 countries) study which enrolled 639 patients with sporadic or familial DCM [8]. Among the enrolled patients, 66% were men, with 49% familial cases and 85% presenting with symptoms of heart failure, similar to our results. The ESC EORP Cardiomyopathy & Myocarditis Registry includes 1260 adult DCM patients (of which 238 have familial DCM) with a similar median age at diagnosis of 49 years old [9]. The mean age of diagnosis in the Romanian cohort was 42 ± 11 years old, according to the typical onset of genetic DCM, with the present cohort being predominantly represented by men, similar to other published studies [8,10,11]. Late-onset DCM has also been described and is more common in women [12]. 

Pathogenic and likely pathogenic variants were identified in 50.8% of the Romanian patients with DCM. The diagnosis yield was higher when compared with results from other studies. Nguyen et al. reported a diagnostic yield of 23% [10], while a study on Finnish patients identified 35% of patients with P and LP variants [11]. This could be explained by more strict selection criteria (such as a younger age and more severe phenotype), considering the relatively limited access to genetic testing in Romania. According to previous observations, the diagnostic yield in familial DCM is higher than that in sporadic cases [11,13]. However, a study on a Chinese cohort that included only people with sporadic dilated cardiomyopathy reported a prevalence of disease-causing variants of 61% [14]. Despite including a lower ratio of familial DCM than other published studies [11,13], our results had a diagnostic yield of 50%, underlying the importance of genetic testing in people with DCM. These results in Romanian people with DCM suggest that, even if a clear familial aggregation cannot be proven, genetic etiology should be investigated. In addition, certain variants may benefit from personalized therapeutic options, thus influencing clinical management. While there are continuous efforts invested in developing molecular-based medicine [15], ICD selection in DCM patients relies on genotype for identifying high-risk variants [1]. Regular follow-ups are advised, even in those with negative phenotypes, because these disorders are often age-dependent. Specific attention is necessary in athletes and pregnant women [16].

Genotype–phenotype correlations have the potential to provide precious information about clinical pictures and cardiovascular outcomes in people with DCM where a disease-causing variant was identified. In concordance with conclusions reported by other authors [8,13,14], we found no significant relation between gender, age at diagnosis, symptoms, LVEF, or arrhythmic risk and positive status. This could be partially explained by the small number of people with a certain gene. DCM severity and evolution have shown important interindividual variability, not only between sporadic cases, but also within individuals of the same family [17]. An explanation could be that clinical phenotype results not only from a single causative gene variant, but also from the interaction with common variants in the genome and epigenetic factors, as well as environmental factors.

Atrial fibrillation was significantly more prevalent in Romanian people with DCM where disease-causing variants were identified compared to those with negative findings. The pathophysiology of atrial fibrillation in genetic DCM is not well understood and the mechanisms overlap. Atrial fibrillation may occur as a consequence of gene-specific defects and/or secondary to structural cardiac architecture changes induced by primary cardiomyopathy [18]. Titin-truncating variants have been shown to directly correlate with atrial fibrillation. Along with ventricular dilation and dysfunction, an important left atrial late gadolinium enhancement has been observed, an indicator of associated atrial fibrosis [19,20]. This induces increased re-entry activity, which may be a possible explanation for the occurrence of atrial fibrillation in these patients. Previous studies described *LMNA* and *SCN5A* variants to increase the risk of atrial fibrillation development, independently to the severity of cardiac dysfunction [21]. These genetic variants were prevalent in our study population.

Truncating disease-causing variants in *TTN* were identified in half of the Romanian people with DCM, consistent with previously published data [8,22]. According to Kayvanpour et al., *TTN* was involved in up to 14–25% of all people with DCM [3], being the most frequently identified disease-causing gene in numerous publications [10,11,23]. DCM caused by *TTN* is associated with an arrhythmic state, both atrial and ventricular, as identified in 50% of the *TTN* group in this study. A total of 537 patients of different ethnicities (317 probands) with truncating variants in the *TTN* gene were followed-up in a study published by Akhtar et al., which showed that male sex and LVEF were independent predictors of adverse outcomes, with no influence between *TTN* variant location and clinical phenotype or prognosis [24]. A significant male predominance was also observed in our study in the *TTN* group compared to the *LMNA* group.

The *LMNA* gene had the second-most prevalent disease-causing variants in our cohort, mostly in female patients. Similarly, *LMNA* variants were identified as being causative for DCM in ∼6–8% of patients by Tayal et al., 2021 [22]. A significant proportion of the variant group had an arrhythmic phenotype (atrial and ventricular) or conduction disorders and device implantation, without statistical significance. According to Taylor et al. [25], the development of atrial fibrillation could be highly suggestive for *LMNA* cardiomyopathy. In our study, 55% of the *LMNA* group had atrial fibrillation. In evolution, this is associated with a poor prognosis [11]. Multiple studies evaluating people with disease-causing variants in *LMNA* have shown rapid disease progression towards advanced cardiac dysfunction and sudden cardiac death through ventricular arrhythmias [26,27,28]. This high arrhythmic risk is correlated with myocardial fibrosis, identified in all the CMR scans of people with *LMNA* variants in our cohort. Disease-causing *LMNA* variants have important clinical and prognostic impacts. Frequent surveillance and adequate early timing for primary prevention ICD implantation are needed, since morbidity and mortality in patients with *LMNA* variants are significant [29].

When comparing the clinical characteristics between the *TTN* versus *LMNA* group in the Romanian patients, the LMNA group presented with significantly more atrio-ventricular conduction abnormalities, device implantation, and atrial fibrillation than the *TTN* group. People with *TTN* variants had a lower rate of fibrosis on CMR, lower BNP levels, and a higher device-free survival curve compared to the *LMNA* group (Figure 2). Overall, this milder expression of the *TTN* group compared to other genes did not reach statistical significance, however, this is in line with the results from the Finish DCM study and others [8,13]. The single case of cardiac transplant from our cohort was in a female with *LMNA*, diagnosed at 18 years old with severe heart failure. The heart transplantation rate was lower in Romanian people with DCM when compared to other European countries [8], possibly because heart transplantation in Romania is less accessible.

The *PLN* gene was typically associated with arrhythmogenic cardiomyopathy [30]. In the Romanian population, *PLN* variants were among the fifth most frequent variants identified in people with DCM, with a particular genetic variant, c.116T>G (p.Leu39Ter), found in three unrelated people, one in a homozygous state. This prevalence is different from the data reported in a large study on people with DCM from eight European countries, where only one variant in the *PLN* gene, c.157_158del (p.53_53del), was described [8]. Possibly, this could be caused by local population characteristics, rather than a common cause of DCM.

Two patients presented a combination of two different variants that could cause DCM. This incidence was lower than the results published by Haas et al., which identified more than one variant in 12% of cases [8]. The association of multiple variants could be responsible for a more severe clinical evolution, as described by Roncarati et al. for double heterozygotes for *LMNA* and *TTN* variant carriers [31]. In this study, one patient with a double heterozygous status for disease-causing variants in *PLN* and *TTN* presented early-age disease onset, an aggressive arrhythmic phenotype, and rapid evolution towards end-stage heart failure. However, the patient from this study with a *PLN* pathogenic variant associated with an *MYBPC3* variant presented a severely impaired cardiac function, yet with a later onset and less arrhythmic phenotype.

In summary, this study shows that NGS can confirm the genetic etiology for an important percentage of patients with DCM. As genetic testing is a dynamic process, with an expanding number of gene phenotype associations being constantly improved, we highlight the need for continuous investigation into genetic determinants in DCM. Thus, the possibility to reinterpret results from sequencing and expand the evaluated genes could bring additional diagnostic yield. Several authors have promoted the use of whole-genome sequencing (WGS) as an alternative to multi-gene panel sequencing for genetic testing in dilated cardiomyopathy. WGS provides a higher variant detection accuracy and the power to identify structural variants. Additionally, WGS offers the opportunity to evaluate disease causality beyond the established disease genes [32,33]. The disadvantages of WGS include, however, a higher cost, increased difficulty in interpretation, and the burden of incidental findings.

The results of genetic testing can change medical management, particularly in a subset of genes that increase the risk for life-threatening ventricular arrhythmias and can influence decisions for defibrillator therapy [16]. The clinical screening and cascade genetic testing of family members should be diligently pursued to identify those at risk of developing DCM [34]. The responsibility of communicating the critical importance of family screening to the patient/family sits with the physician, preferably in conjunction with an expert genetics team, in order to facilitate cascade genetic testing or broad clinical family screening.

### Study Limitations

This study relied on clinical data provided by ordering providers. In contrast to a controlled clinical study in which all patients are evaluated using a common set of diagnostic criteria, our clinical data are likely more heterogeneous. A second limitation is that, due to the expanding size of our test panels over time, not all genes were tested in all cases, and therefore, gene-specific detection rates are representative for the number of patients tested, rather than for the entire cohort. However, all patients had a core set of 54 genes associated with DCM. Non-coding deep intronic variants were not evaluated in this cohort, although reports have shown the possible role of non-coding variants [35]. At the time of writing the manuscript, to our knowledge, this is the largest reported analysis of genetic variation in patients with DCM from Romania. However, it might not be large enough to be immune to statistical fluctuations that commonly afflict small- to medium-sized cohorts [36]. Family screening information was limited in this cohort, possibly leading to underreporting on affected family members.

## 4. Materials and Methods

### 4.1. Patient Evaluation

The study cohort consisted of 122 patients with a diagnosis of DCM who were followed at five tertiary University Hospitals in Romania (Bucharest, Timișoara, Craiova, Iași, Cluj-Napoca) and received genetic testing. The study included patients with genetic testing for DCM etiology evaluated between September 2016 and December 2022. The clinical data of the index patients were collected retrospectively from the time of their DCM diagnosis. DCM was diagnosed according to the following criteria: left ventricular end-diastolic (LVED) volumes or diameters above the established cut-off according to gender and corrected by body surface area (BSA) and left ventricular ejection fraction (LVEF) of <50%, in absence of high loading conditions (e.g., significant valvulopathies or congenital heart disorders) or significant coronary artery disease [1,37]. Before enrollment, myocardial ischemia was excluded using a coronary multislice computed tomography angiography or a coronary artery angiography according to the local resources available. Patients with history of cardiotoxic treatment, chronic alcohol consumption, those with clinical suspicion of myocarditis, and those with syndromic forms of DCM were excluded. All criteria had to be fulfilled at the time of the initial DCM diagnosis in the patient. The following information was obtained for each individual during the evaluation: (a) symptoms of cardiac disease; (b) results of electrocardiogram (ECG) and Holter-recordings; (c) results of echocardiography; (d) cardiac magnetic resonance (CMR) characterization, (e) implantation of a cardiac device: pacemaker, implantable cardioverter-defibrillators (ICD), Cardiac Resynchronization Therapy Devices (CRTD), or cardiac resynchronization therapy with pacemaker (CRTP); (f) disease complications, including significant ventricular arrhythmias (VA) (e.g., sustained or non-sustained ventricular tachycardia and history of resuscitated cardiac arrest) or heart transplants, and (g) all-cause mortality. Family history was evaluated in each index patient using a 3–4 generations pedigree. Familial DCM was defined as the presence of more than one affected individual (deceased or alive) with a confirmed diagnosis of DCM or SCD. All relatives at risk of having inherited the condition were offered clinical investigations, consisting of a physical examination, echocardiography, and ECG. Relatives with normal clinical investigations were offered a prospective follow-up every 3 or 5 years. The family members identified with the causative variant with cardiomyopathy were included in the study, if phenotype-positive. Sporadic DCM was defined in the absence of a significant family history of DCM or SCD. The DCM patients were followed yearly according to standard guidelines. A cut-off of 35% for LVEF was used in the analysis, considering that primary prevention recommendations consider implanting an ICD in people with DCM and LVEF of ≤35% [1].

### 4.2. Molecular Diagnostic Tests for DCM

Based on availability and local collaboration protocols, for some of the patients, NGS gene panels were used to test for sequence- and exon-level copy number variants at the Invitae laboratories (USA) previously described [38,39]. At the prescribing physician’s discretion, one panel among various commercially available NGS panels was selected (e.g., dedicated to DCM, comprehensive cardiomyopathy panels, and cardiomyopathy and arrhythmia panels). The basic commercial panel included 54 genes with established associations with DCM (Invitae Dilated Cardiomyopathy and Left Ventricular Non-compaction Panel). Larger panels up to 179 genes included a broader set of genes for cardiomyopathies and sudden cardiac death genes, such as the Invitae Arrhythmia Comprehensive Panel, Invitae Dilated Cardiomyopathy and Left Ventricular Noncompaction Panel, Invitae Cardiomyopathy Comprehensive Panel, and Invitae Arrhythmogenic Cardiomyopathy Panel. Familial screening was offered to relatives of a proband with pathogenic, likely pathogenic, and variants of unknown significance. Screening was performed by sequencing the significant genes found in the related proband (Invitae).

Other people with DCM had genetic testing performed in the Genomic Center of the University of Medicine and Pharmacy Victor Babes Timisoara, using a sequencing panel of 174 genes, the TruSightCardio panel Illumina (San Diego, CA, USA). Target enrichment was performed with the TruSight Rapid Capture kit (Illumina). Captured libraries were sequenced with 2 × 150 bp reads on a MiSeq platform (Illumina). Sequence reads were mapped onto the human reference genome, hg37, using the Burrows–Wheeler alignment (BWA) tool. The identified variants were annotated using ANNOVAR, as previously published [40,41]. The patient and physician, considering different turnaround times and reimbursements, opted between the two laboratories.

### 4.3. Variant Interpretation Criteria

The results were interpreted in the contexts of population, segregation, computation, and the functional data available. The databases of human genetic variation used included the Genome Aggregation Database (gnomAD version 3.1; http://gnomad.broadinstutite.org, accessed on 5 August 2023), VarSome (https://varsome.com/ VarSome 11.8, accessed on 5 August 2023) [35], and ClinVar (https://www.ncbi.nlm.nih.gov/clinvar, accessed on 5 August 2023) [42]. The Clinical Genome Resource (ClinGen; https://www.clinicalgenome.org/ accessed 5 September 2023) [43] was used for expertly curated reports of clinically relevant genes and their variants. The American College of Medical Genetics and Genomics and the Association for Molecular Pathology (ACMG/AMP) was used to classify a genetic variant as benign (B), likely benign (LB), a variant of uncertain significance (VUS), likely pathogenic (LP), or pathogenic (P) [23]. The results were categorized as disease-causing/significant (if P or LP), negative (if B or LB), or uncertain (VUS), depending on the classification of the variant identified and the inheritance pattern of the associated condition. Carrier status for autosomal recessive disorders, identified in the present cohort, is not discussed in this article.

### 4.4. Statistical Analysis

Continuous variables (normally distributed) were expressed as mean and standard deviation (SD), while non-parametric variables were expressed as median (interquartile range). Categorical variables were presented as numbers (proportions). Independent sample t-tests combined with Levene’s tests were used for comparison between groups of continuous variables, and Mann–Whitney U-tests were used for non-parametric variables. Categorical variables were compared using the chi-squared test. The Kaplan–Meier curve was used for device-free survival analyses. The P values for the comparison between curves in patients with versus without significant genetic variants identified, male versus female, *TTN* group versus other genes, and *TTN* group versus *LMNA* group were calculated using Log Rank (Mantel–Cox test). Statistical significance was set at a 2-sided *p*-value of <0.05. SPSS 12.0 (IBM SPSS Inc. Chicago, IL, USA) was used for statistical analysis.

### 4.5. Ethics Statement

This retrospective study was conducted in accordance with the Declaration of Helsinki and approved by the local ethics committee (no. 60/15 December 2017 from the University of Medicine and Pharmacy ‘Victor Babeș’ Timișoara, and no. 17748/24 June 2022 from University of Medicine and Pharmacy ‘Carol Davila’, Bucharest). Informed consent for genetic testing and the research study was obtained from all participants of the study.

### 4.6. Declaration of Generative AI and AI-Assisted Technologies in the Writing Process

The authors did not use generative AI or AI-assisted technologies in the development of this manuscript.

## 5. Conclusions

The significant percentage of pathogenic/likely pathogenic variants identified in Romanian patients with DCM shows the importance of genetic investigations. This study shows the genetic landscape in genes associated with DCM in Romanian people. Variants in *TTN*, *LMNA*, and *DSP* explained 75% of the genetic causes for DCM from Romania. Significant differences in clinical presentation were not observed between those with disease-causing variants and negative findings in genetic testing, supporting that we cannot anticipate the presence of a causative variant based only on clinical picture.

Lay summary:

Dilated cardiomyopathy (DCM) leads to risk of heart failure and sudden cardiac death, and frequently involves a genetic etiology. Genetic testing is valuable for risk stratification, treatment decisions, and family screening. Romanian population data on the genetic etiology of DCM are lacking. We aimed to investigate the genetic causes for DCM among Romanian adult patients. The substantial percentage of disease-causing variants identified highlights the genetic landscape of DCM in Romanian people, including 31 novel variants, to the best of our knowledge.

Clinical implications:

- Genetic investigations should be considered in sporadic DCM, considering that disease-causing variants were identified in a substantial percentage in this group.
- Family screening, including clinical evaluation and subsequent genetic testing, should be offered to all relatives of index patients with confirmed familial DCM.

## Figures and Tables

**Table 1:** Clinical characteristics of people with DCM, comparison between people with disease-causing versus variants of unknown significance and negative findings in genetic testing, and comparison between familial and sporadic cases.

Variable	All n = 122	Disease-Causing Findings n = 62	Negative & VUS n = 60	*p* Value	Familial n = 64	Sporadic n = 58	*p* Value
Gender male n (%)	79 (66.6%)	41 (66.6%)	39 (65.5%)	0.524	35 (54.6%)	45 (77.6%)	**0.006**
Age, years (mean ± SD)	45.8 ± 12.8	44.9 ± 13.1	46.8 ± 12.4	0.415	43.5 ± 12.0	48.3 ± 13.2	**0.039**
Age at diagnosis (mean ± SD)	41.4 ± 12.3	40.2 ± 12.5	42.7 ± 12.1	0.277	38.7 ± 11.4	44.4 ± 12.8	**0.011**
**Presentation reason n (%)**							
Cardiac symptoms	101 (82.3%)	48 (80.0%)	53 (88.3%)	0.191	48 (75%)	53 (91.4%)	**0.011**
Sudden cardiac death episode	3 (2.4%)	2 (3.2%)	1 (1.7%)	0.512	1 (1.5%)	2 (3.4%)	0.463
Incidental discovery	8(6.5%)	3 (4.8%)	5(8.3%)	0.389	4 (6.2%)	4 (6.9%)	**0.011**
Family screening	10 (8.1%)	8 (12.9%)	2(3.3%)	0.122	10 (15.6%)	0	**0.012**
**Heart failure features**							
LVEF (mean ± SD)	35.3 ± 11.1	36.2 ± 11.7	34.3 ± 10.4	0.343	36.6 ± 10.9	33.8 ± 11.2	0.168
LVEF ≤ 35% n (%)	59 (48.3%)	26 (41.9%)	33 (55%)	0.103	28 (43.7%)	31 (53.4%)	0.187
NTproBNP (pg/mL) n = 63	3357.1 ± 442.4	3967.09 ± 4971.13	2642.03 ± 3649.22	0.062	3053.86 ± 4029.21	3653.86 ± 4853.17	0.126
BNP (pg/mL) n = 39	435.6 ± 744.7	662.9 ± 974.0	219.7 ± 331.1	0.073	275.2 ± 554.7	722.0 ± 956.6	0.072
**Complications n (%)**							
Ventricular arrhythmias	35 (28.6%)	20 (32.2%)	15 (25%)	0.407	17 (26.5%)	20 (34.5%)	0.204
Atrial fibrillation	23 (18.8%)	16 (25.8%)	7 (11.6%)	**0.041**	12 (18.7%)	11 (18.9%)	0.599
Heart conduction disorder	41 (33.3%)	17 (27.4%)	24 (40%)	0.159	19 (29.7%)	22 (37.9%)	0.175
Fibrosis on CMR	54/73 (73.9%)	31/41 (75.6%)	23/32 (71.8%)	0.461	30/38 (78.9%)	24/35 (80.6%)	0.229
Heart transplantation	1 (0.8%)	1 (1.6%)	0	0.529	1 (1.6%)	0	0.529
Deceased	5 (4.1%)	3 (4.8%)	2 (3.3%)	0.508	4 (6.2%)	1 (1.7%)	0.215
**Devices**							
Pacemaker n (%)	6 (4.9%)	3 (4.8%)	3 (5%)	0.644	3 (4.6%)	3 (5.2%)	0.612
Any ICD (ICD + CRT-D) n (%)	36 (29.3%)	22 (35.4%)	14 (23.3%)	0.124	21 (32.8%)	15 (25.8%)	0.352
CRTP n (%)	6 (4.9%)	2 (3.2%)	4 (6.6%)	0.324	1 (1.6%)	5 (86.2%)	0.083
Age at ICD implant n = 35 (mean ± SD)	40.97 ± 13.58	39.29 ± 13.53	43.50 ± 13.74	0.379	39.40 ± 13.41	43.07 ± 13.98	0.441
Age at CRT implant n = 14 (mean ± SD)	49.43 ± 13.09	50.20 ± 6.79	49.00 ± 15.96	0.877	47.75 ± 19.19	50.10 ± 11.09	0.830

Legend: number (n), Left Ventricle Ejection fraction (LVEF); implantable cardioverter-defibrillators (ICD); Cardiac Resynchronization Therapy Devices (CRTD); cardiac resynchronization therapy with pacemaker (CRTP); MRI magnetic resonance imaging; and variants of unknown significance (VUS).

**Table 2:** Pathogenic and likely pathogenic variants in genes (alphabetic gene order).

Familial or Sporadic	Sex	Age at Diagnosis (Years)	Gene	NM Number	Transcript (Protein)	Zygosity	Classify-Cation	Variant Type
familial	M	32	*ACTC1*	NM_005159.5	c.854T>A (p.Met285Lys)	het	LP (VUS)	missense *
familial	M	47	*ACTC1*	NM_005159.5	c.854T>A (p.Met285Lys)	het	LP (VUS)	missense *
familial	M	39	*BAG3*	NM_004281.4	c.920dupC (p.H308Pfs)	het	LP	frameshift *
sporadic	M	56	*CRYAB*	NM_001289808.2	c.166C>T (p.Arg56Trp)	het	LP	missense
familial	M	17	*DMD*	NM_004006.3	c.10801C>T (p.Gln3601Ter)	het	P	nonsense *
sporadic	M	20	*DMD*	NM_004006.3	c.6913-?_7200+?del	het	P	frameshift
sporadic	F	41	*DSP*	NM_004415.4	c.(2630+1_2631-1)_(2877+2878-1)del	het	LP	frameshift
sporadic	F	51	*DSP*	NM_004415.2	c.2498dup (p.Lys834Glufs)	het	P	frameshift
familial	F	38	*DSP*	NM_004415.2	c.313C>T (p.Arg105Ter)	het	P	nonsense
familial	M	42	*DSP*	NM_004415.2	c.313C>T (p.Arg105Ter)	het	P	nonsense
sporadic	F	41	*DSP*	NM_004415.2	c.5212C>T (p.Arg1738Ter)	het	P	nonsense
sporadic	M	70	*DSP*	NM_004415.2	c.597+1G>A	het	LP	splicesite
familial	F	50	*LMNA*	NM_170707.4	c.1003C>T (p.Arg335Trp)	het	P	missense
familial	M	28	*LMNA*	NM_170707.4	c.1003C>T p.(Arg335Trp)	het	P	missense
familial	F	19	*LMNA*	NM_170707.3	c.448A>C (p.Thr150Pro)	het	P	missense
familial	F	16	*LMNA*	NM_170707.3	c.448A>C (p.Thr150Pro)	het	LP	missense
familial	F	44	*LMNA*	NM_170707.3	c.604G>T (p.Glu202Ter)	het	P	nonsense
sporadic	F	45	*LMNA*	NM_170707.3	c.673C>T (p.Arg225Ter)	het	P	nonsense
sporadic	M	68	*LMNA*	NM_170707.3	c.886C>T (p.Arg296Cys)	het	LP (VUS)	missense
familial	F	40	*LMNA*	NM_170707.3	c.980_995del (p.Leu327Profs)	het	P	frameshift
familial	F	41	*LMNA*	NM_170707.3	c.980_995del (p.Leu327Profs)	het	P	frameshift
^$^ sporadic	M	64	*MYBPC3*	NM_000256.3	c.1504C>T (p.Arg502Trp)	het	P	missense
sporadic	M	24	*MYH7*	NM_000257.4	c.2458G>A (p.Ala820Thr)	het	LP	missense *
sporadic	M	31	*MYO6*	NM_004999.3	c.755G>A (p.Cys252Tyr)	het	LP (VUS)	missense *
^$^ sporadic	M	64	*PLN*	NM_002667.3	c.116T>G (p.Leu39Ter)	het	P	nonsense
^#^ sporadic	M	44	*PLN*	NM_002667.3	c.116T>G (p.Leu39Ter)	het	P	missense
familial	F	30	*PLN*	NM_002667.3	c.116T>G (p.Leu39Ter)	hom	P	missense
familial	M	31	*RBM20*	NM_001134363.2	c.1913C>T (p.Pro638Leu)	het	P	missense
sporadic	M	34	*RBM20*	NM_001134363.3	c.2737G>A (p.Glu913Lys)	het	P	missense
^ familial	M	20	*RYR1*	NM_000540.2	Deletion (Exons 48-49)	het	P	frameshift
sporadic	M	27	*TMEM43*	NM_024334.2	c.718C>A (p.Arg240Ser)	het	LP (VUS)	missense *
familial	F	49	*TNNT2*	NM_001001430.2	c.400C>T (p.Arg134Trp)	het	LP	missense
sporadic	M	42	*TNNT2*	NM_001001430.2	c.517C>T (p.Arg173Trp)	het	LP	missense
familial	F	30	*TTN*	NM_001267550.2	c.101418del (p.Ala33807HisfsTer)	het	LP	frameshift *
sporadic	M	40	*TTN*	NM_001267550.2	c.101793_101794del (p.His33931GlnfsTer)	het	LP	frameshift
familial	M	31	*TTN*	NM_001267550.2	c.107635C>T (p.Gln35879Ter)	het	LP	nonsense
sporadic	M	65	*TTN*	NM_001267550.2	c.46986dup (p.Asn15663Ter)	het	P	frameshift *
^ familial	M	20	*TTN*	NM_001267550.2	c.46986dup (p.Asn15663Ter)	het	P	frameshift *
familial	F	65	*TTN*	NM_001267550.2	c.60733C>T (p.Arg20245Ter)	het	P	nonsense
familial	M	52	*TTN*	NM_001267550.2	c.60733C>T (p.Arg20245Ter)	het	P	nonsense
familial	F	45	*TTN*	NM_001267550.2	c.68269del (p.His22757ThrfsTer)	het	LP	frameshift *
familial	M	45	*TTN*	NM_001267550.2	c.68575_68576dup (p.Ile22861SerfsTer)	het	LP	frameshift *
familial	M	50	*TTN*	NM_001267550.2	c.68575_68576dup (p.Ile22861SerfsTer)	het	LP	frameshift *
sporadic	M	41	*TTN*	NM_001267550.2	c.68824del (p.Glu22942ArgfsTer)	het	LP	frameshift
sporadic	M	55	*TTN*	NM_001267550.2	c.70437del (p.Lys23480AsnfsTer)	het	LP	frameshift *
familial	M	39	*TTN*	NM_001267550.2	c.70437del (p.Lys23480AsnfsTer)	het	LP	frameshift *
sporadic	M	32	*TTN*	NM_001267550.2	c.70978C>T (p.Arg23660Ter)	het	P	nonsense
familial	M	33	*TTN*	NM_001267550.2	c.73646C>G (p.Ser24549Ter)	het	LP	nonsense *
sporadic	F	54	*TTN*	NM_001267550.2	c.73646C>G (p.Ser24549Ter)	het	LP	nonsense *
sporadic	M	36	*TTN*	NM_001267550.2	c.74338C>T (p.Arg24780Ter)	het	P	nonsense
familial	M	39	*TTN*	NM_001267550.2	c.74338C>T (p.Arg24780Ter)	het	P	nonsense
familial	F	55	*TTN*	NM_001267550.2	c.79273A>T (p.Lys26425Ter)	het	LP	nonsense *
familial	F	34	*TTN*	NM_001267550.2	c.79273A>T (p.Lys26425Ter)	het	LP	nonsense *
familial	M	28	*TTN*	NM_001267550.2	c.82172G>A (p.Trp27391Ter)	het	LP	nonsense *
familial	F	48	*TTN*	NM_001267550.2	c.82172G>A (p.Trp27391Ter)	het	LP	nonsense *
familial	M	47	*TTN*	NM_001267550.2	c.82415_82419dup (p.Ser27474LeufsTer)	het	LP	frameshift *
familial	M	46	*TTN*	NM_001267550.2	c.82415_82419dup (p.Ser27474LeufsTer)	het	LP	frameshift *
familial	F	35	*TTN*	NM_001267550.2	c.89882_89885del (p.Gly29961AspfsTer)	het	LP	frameshift *
sporadic	M	32	*TTN*	NM_001267550.2	c.92294del (p.Arg30765AsnfsTer3)	het	LP	frameshift *
sporadic	M	21	*TTN*	NM_001267550.2	c.92317C>T (p.Arg30773Ter)	het	P	nonsense
sporadic	M	33	*TTN*	NM_001267550.2	c.94996_95008del (p.Tyr31666GlufsTer)	het	LP	frameshift *
sporadic	M	36	*TTN*	NM_001267550.2	c.95055del (p.Lys31685AsnfsTer4)	het	LP	frameshift *
familial	M	43	*TTN*	NM_001267550.2	c.59626+1G>A	het	LP	splicesite *
familial	M	43	*TTN*	NM_001267550.2	c.71184del (Pro23729HisfsTer16)	het	LP	frameshift *
^#^ sporadic	M	44	*TTN*	NM_001267550.2	c.94128del (p.Lys31375_Tyr31376insTer)	het	LP	frameshift *

Novel variants (to the best of our knowledge) are marked with *, F = Female, M = male, het = heterozygous, and hom = homozygous; in grey: ^#,^,$^ are mare makings for individuals with more than one variant identified.

**Table 3:** Clinical characteristics of people with DCM and disease-causing genetic findings—comparison between *TTN* versus other genes and between *TTN* and *LMNA* genes.

Variable	All Positive n = 62	TTN n = 32	Other Genes n = 30	*p* Value TTN/Other	LMNA n = 9	*p* Value TTN/LMNA
Gender male n (%)	41 (66.1)	24 (75%)	17 (56.7%)	0.104	2 (22.2%)	**0.006**
Age, years (mean ± SD)	44.9 ± 13.1	45.1 ± 11.6	44.7 ± 14.8	0.915	44.1 ± 17.3	0.847
Age at diagnosis (mean ± SD)	40.2 ± 12.6	41.2 ± 10.9	39.2 ± 14.1	0.550	39.0 ± 16.1	0.880
**Presentation reason n (%)**						
Cardiac symptoms	49 (79%)	24 (75%)	25 (83.3%)	0.679	8 (88.9%)	0.350
Sudden cardiac death episode	2 (3.2%)	1 (3.1%)	1 (3.3%)	0.738	0	0.780
Incidental discovery	4 (6.4%)	2 (6.2%)	2 (6.6%)	0.910	0	0.427
Family screening no symptoms	8 (12.9%)	5 (15.6%)	3 (10.0%)	0.645	1 (1.1%)	0.176
**Heart failure**						
LVEF value (mean ± SD)	36.2 ± 11.7	34.9 ± 11.7	37.6 ± 11.7	0.359	41.9 ± 9.2	0.073
LVEF ≤ 35% n (%)	26 (41.9%)	15 (46.9%)	11 (36.3%)	0.289	2 (22.2%)	0.174
NTproBNP (pg/mL) (mean ± SD)	3967.1 ± 4971.0 (n = 34)	3695.3 ± 5083.2 (n = 21)	4406.0 ± 4955.4 (n = 13)	0.691	5533.6 ± 6256.4 (n = 6))	0.464
BNP (pg/mL) (mean ± SD)	662.8 ± 974.0 (n = 19)	33.5 ± 41.7 (n = 4)	830.6 ± 1037.3 (n = 15)	**0.010**	659.0 ± 729.5 (n = 4)	0.185
**Complications n (%)**						
Ventricular arrhythmias	17 (27.4%)	9 (28.1%)	3 (10%)	0.293	4 (44.4%)	0.294
Atrial fibrillation	16 (25.8%)	7 (21.9%)	9 (30%)	0.301	5 (55.6%)	0.064
Heart conduction disorder	17 (27.4%)	6 (18.8%)	11 (36.7)	0.097	6 (66.7%)	**0.011**
Fibrosis on MRI	31/41 (76.5%)	16/21 (76.2%)	16/20 (80.0%)	0.463	8/8 (100%)	0.126
Heart transplantation	1 (1.6%)	0	1 (3.3%)	0.475	1 (11.1%)	0.220
Deceased	3 (4.8%)	2 (6.3%)	1 (3.3%)	0.525	0	0.605
**Devices**						
Pacemaker n (%)	3 (4.8%)	1 (3.1%)	2 (6.7%)	0.475	1 (11.1%)	0.395
Any ICD (ICD + CRT-D) n (%)	22 (35.5%)	9 (28.1%)	13 (43.3%)	0.115	6 (66.7%)	**0.022**
CRTP n (%)	3 (4.8%)	1	2 (6.7)	0.230	1 (11.1%)	0.220
Age at ICD implant (mean ± SD)	39.3 ± 13.5	42.22 ± 13.49	37.1 ± 13.7	0.403	33.3 ± 14.2	0.243
Age at CRT implant (mean ± SD)	50.0 ± 6.8	50	50.2 ± 7.8	0.979	52	-

## Data Availability

Data is contained within the article and Supplementary Material.

## Supplementary Materials

The following supporting information can be downloaded at: https://www.mdpi.com/article/10.3390/ijms25052562/s1.

## Abbreviations

ACMG	The American College of Medical Genetics and Genomics
AMP	Association for Molecular Pathology
B	Benign
BNP	brain natriuretic peptides
BSA	body surface area
BWA	Burrows–Wheeler alignment
CMR	cardiac magnetic resonance
CRTD	Cardiac Resynchronization Therapy Devices
CRTP	cardiac resynchronization therapy with pacemaker
DCM	dilated cardiomyopathy
ECG	Electrocardiogram
HF	heart failure
ICD	implantable cardioverter-defibrillator
LB	likely benign
LP	likely pathogenic
LVED	left ventricular end-diastolic
LVEF	left ventricular ejection fraction
NGS	next-generation sequencing
P	Pathogenic
SCD	sudden cardiac death
SD	standard deviation
VUS	variant of uncertain significance
WGS	whole-genome sequencing
